# Myxomatous Mitral Valve Disease in Large Breed Dogs: Survival Characteristics and Prognostic Variables

**DOI:** 10.3390/vetsci11030136

**Published:** 2024-03-21

**Authors:** Mikael Svensson, Jonas Selling, Mark Dirven

**Affiliations:** 1 Evidensia Valla Djursjukhus, 582 13 Linköping, Sweden; 2 Statistikakademin, 753 21 Uppsala, Sweden; jonas.selling@statistikakademin.se; 3 Evidensia Södra Djursjukhuset, 141 75 Stockholm, Sweden; mark.dirven@evidensia.se

**Keywords:** dog, heart, mitral valve disease, echocardiography, ACVIM

## Abstract

**Simple Summary:**

Myxomatous mitral valve disease (MMVD) is the most commonly acquired heart disease in dogs. It is mainly diagnosed in small-and medium-sized dogs but large breed dogs can be affected as well. MMVD can, in some cases, lead to heart failure and an early death. The aim of this study was to describe the natural course of the disease in large breed dogs to try and fill some of the knowledge gaps that exist. Medical records of dogs diagnosed with MMVD, between 2012 and 2021, were retrospectively reviewed and 202 dogs were included in the analysis. Our results suggest that the frequency of cardiac-related death is low in large breed dogs with MMVD and a normal heart size. However, the risk of an unfavorable outcome escalates significantly as the left atrium and ventricle enlarge, and the dog progresses from one stage of the disease to another.

**Abstract:**

Myxomatous mitral valve disease (MMVD) is the most common acquired heart disease in dogs and its occurrence in small-and medium-sized dogs has been extensively investigated. MMVD has been described in large breed dogs as well, but substantial knowledge gaps remain. The aim of this study was to provide characteristics, survival times, and prognostic variables in large breed dogs with MMVD. Medical records of dogs diagnosed with MMVD, between 2012 and 2021, were retrospectively reviewed and 202 dogs were analyzed. Median survival time (MST) for all-cause mortality was 800 days for stage B1 dogs, 274 days for stage B2 dogs, and 184 days for stage C dogs. The MST for cardiac-related death for B1 dogs could not be calculated (because survival was greater than 50% at the last timepoint) and for stage B2 and C dogs the MST were 484 and 252 days, respectively. These findings suggest that the frequency of cardiac-related death is low in large breed dogs with stage B1 MMVD. In addition, increased left atrial and ventricular size, evidence of systolic dysfunction, a thrilling murmur, and increased early trans-mitral peak velocity are predictors of cardiac-related death. Data also suggest that the risk of a negative outcome increases profoundly when large breed dogs advance from ACVIM stage B1 into stage B2 or C.

## 1. Introduction

Myxomatous mitral valve disease (MMVD) is the most common acquired heart disease in dogs [1,2,3,4,5,6]. MMVD is characterized by chronic and slowly progressive valvular changes and is most often reported in small-and medium-sized, middle-aged to older dogs, that typically present with an incidental left apical systolic murmur [3,4,7,8]. The progressive nature of the disease results, in a subset of dogs, in eccentric left ventricular hypertrophy, left atrial enlargement, and eventually in left-sided congestive heart failure, which are associated with significant morbidity and mortality despite medical intervention [2,7,8,9].

Why dogs develop MMVD is largely unknown, but it has been found to have an inherited component in breeds such as Dachshund and Cavalier King Charles Spaniel (CKCS) [10,11,12,13].

Although most frequently diagnosed in small- and medium-sized dogs, MMVD also occurs in larger dogs [5,7,14,15,16]. However, MMVD in large breed dogs seems to present somewhat differently than in their small breed counterparts. In a retrospective study comparing German Shepherd dogs (GSD) to small- and medium-sized dogs (<15 kg) with MMVD, it was found that GSD with MMVD developed atrial fibrillation more frequently, had less pronounced echocardiographic valvular lesions, and had evidence of left ventricular systolic dysfunction [14].

In the ACVIM consensus guidelines for the diagnosis and treatment of MMVD in dogs, some panelists recommend more frequent echocardiographic follow up in the preclinical large breed dog with MMVD as it has been stated that MMVD in larger breeds progresses faster and has worse prognosis than in their small breed counterparts [6].

Although the natural history of MMVD in large breed dogs has been addressed in several publications, substantial gaps remain, including survival times and prognostic variables specific to this group of dogs [5,7,14,15,16]. There appears to be very little data supporting the belief that MMVD in large breed dogs may progress faster, with systolic dysfunction and worse prognosis [6,16].

To be able to extrapolate results from high quality studies based on small-and medium-sized dogs, one need to know if MMVD behaves in the same way in large breed dogs. If the two populations do not respond in the same way, it underscores the need for further research and careful consideration when planning future studies.

Therefore, the aim of this study is to provide characteristics, estimated survival times and prognostic variables in a cohort of large breed dogs (≥20 kg) with naturally occurring MMVD.

## 2. Materials and Methods

### 2.1. Animals

Medical records of dogs diagnosed with MMVD at Anicura Regiondjursjukhuset Bagarmossen (ARB) and Anicura Albano Djursjukhus (AAD) between 2012 and 2021 were reviewed. Cases were referred to the cardiology service either from external primary care clinics, that rarely perform echocardiographic examinations in Sweden, or internally from other departments. Most of the cases were referred based on auscultation of a cardiac murmur.

### 2.2. Inclusion Criteria

As most studies only include dogs with a weight of up to 20 kg, this study only included dogs that weighed 20 kg or more [8,9,17,18,19,20,21]. In addition, they had to be older than 5.5 years and diagnosed with MMVD based on the presence of a heart murmur and one or more of the following echocardiographic findings: mitral valve prolapse, color doppler identification of any degree of mitral valve regurgitation, or mitral valve thickening [7,8,20].

The following clinical data had to be available from the medical records for a dog to be included in the study: age (if only year (yy) was provided in the medical record, the dog was assigned 15/06/yy and if only month and year (mm/yy) was provided the dog was assigned 15/mm/yy), sex, bodyweight (if no data were available from the date of diagnosis, closest known data within one year or if possible an average of the first weight recorded before and after diagnosis, were used), and breed.

The following echocardiographic data had to be available: left ventricular internal diameter in diastole (LVIDd), left ventricular internal diameter in systole (LVIDs), fractional shortening (FS), and left atrial-to-aortic ratio (LA/Ao) measured on the first frame after aortic valve closure [22].

### 2.3. Exclusion Criteria

Exclusion criteria were as follows: incomplete clinical or echocardiographic data, age equal to or below 5.5 years, FS equal to or below 19.5%, a diagnosis of congenital heart disease, endocarditis, cardiac neoplasia, dilated cardiomyopathy (DCM), uncertain diagnosis or changes in diagnosis over time (e.g., changing from suspected MMVD to DCM or vice versa), contradictory or erroneous data (e.g., incorrectly placed decimal points, typing errors, contradictory values of left ventricular dimensions and FS), and outdated record of bodyweight (more than one year from diagnosis). In addition, dogs were excluded if cause and date of death were not available from the medical records or from a conducted telephone interview (some owners chose, however, to respond through email).

### 2.4. Additional Data

In addition to set inclusion and exclusion criteria, the following data were also recorded when available: murmur grade was recorded and classified as soft (I–II), moderate/loud (III–IV), or thrilling (V–VI) [23]. The moderate and loud subgroups were merged because of the difference in grading systems used in the medical records. Some dogs were graded according to the 6-level scheme by Levine (1933), while others were graded according to a modified version (low (I–II), medium (III–IV), and high (V–VI), used by Häggström et al. (1995) [24,25]. Furthermore, atrial fibrillation (yes/no) and any ongoing cardiac treatment at the time of diagnosis were recorded.

Echocardiographic examinations were performed by veterinarians experienced in echocardiography including European and Swedish specialists in cardiology and veterinarians in training programs to become specialists in cardiology (residents or national specialization track). The echocardiographic measurements from the original examinations were used for data analysis.

LVIDd and LVIDs were normalized to bodyweight and every included dog was staged by one of the authors (M.S.) according to the current American College of Veterinary Internal Medicine (ACVIM) consensus guidelines for the diagnosis and treatment of myxomatous mitral valve disease in dogs [6,26]. As radiographs and exact murmur grade were not available for every dog in this retrospective study, staging relied solely on echocardiographic parameters.

Stage B1: echocardiographic LA/Ao ratio < 1.6 and/or LVIDDN < 1.7. Stage B2: echocardiographic LA/Ao ≥ 1.6 and LVIDDN ≥ 1.7. Stage C: B2 + clinical evidence of congestive heart failure.

Congestive heart failure was diagnosed through compatible clinical signs and detected radiological changes consistent with pulmonary oedema. Radiographs were evaluated by the clinician responsible for the case or a specialist in diagnostic imaging. Stage D: dogs that require more than a total daily dosage of 8 mg/kg of furosemide in addition to standard therapy to control clinical signs of heart failure [6]. Early trans-mitral peak velocity (MVE) was recorded if available but was not a definitive inclusion criterion.

Furthermore, if additional echocardiographic examinations were available in the medical records, data from the last available echocardiographic follow-up examination were recorded and obtained measured and/or calculated data included date of follow-up examination, LVIDd, LVIDDN, LVIDs, LVIDSN, LA/Ao, FS, MVE, and ACVIM stage.

### 2.5. Follow-Up

Cases were followed until 31 December 2021. The primary endpoints of the study were all-cause mortality and cardiac-related death. Cause and date of death were either retrieved from the medical records or a telephone interview was conducted with the owner (some owners chose, however, to respond through email). Death was classified as either spontaneous or through euthanasia. Reason for euthanasia or spontaneous death was recorded.

The cause of euthanasia or spontaneous death was classified as cardiac-related if death occurred because of progression of clinical signs of heart disease, if the dog was found dead and had been apparently well during the preceding 24 h or, if death was witnessed, and the dog had no apparent signs of disease during the preceding hour [27,28]. If cause of death was uncertain, it was classified as non-cardiac.

### 2.6. Statistical Analysis

In the study, two dependent variables (DV) were used, listed as follows: survival for all-cause mortality and survival for cardiac-related death. A total of 11 independent (IV) variables were used, listed as follows: age, sex, bodyweight, murmur grade, LVIDDN, LVIDSN, LVIDSN >1.26, LA/Ao, FS, MVE, and ACVIM stage.

All descriptive statistics for categorical variables are shown as frequencies and percentages and for continuous variables, as mean and standard deviation (SD). Kaplan–Meier estimates were used to graphically show the survival curves. Survival times are reported as median and 95 percent confidence interval (CI).

The Cox proportional hazards model was used to calculate hazard ratios (HR), 95 percent CI, and corresponding *p*-values for both DV. Each IV was firstly modeled alone to obtain the unadjusted results for HR, CI, and *p*-values, and then modeled together with age, sex, and bodyweight to obtain the adjusted results. Influence of outliers and proportional hazard was checked for all Cox models. A *p*-value < 0.05 was considered significant.

Statistical analyses were carried out using a commercially available software program (IBM SPSS Statistics version 28.0.1.1 (14)).

## 3. Results

**Figure 1:**
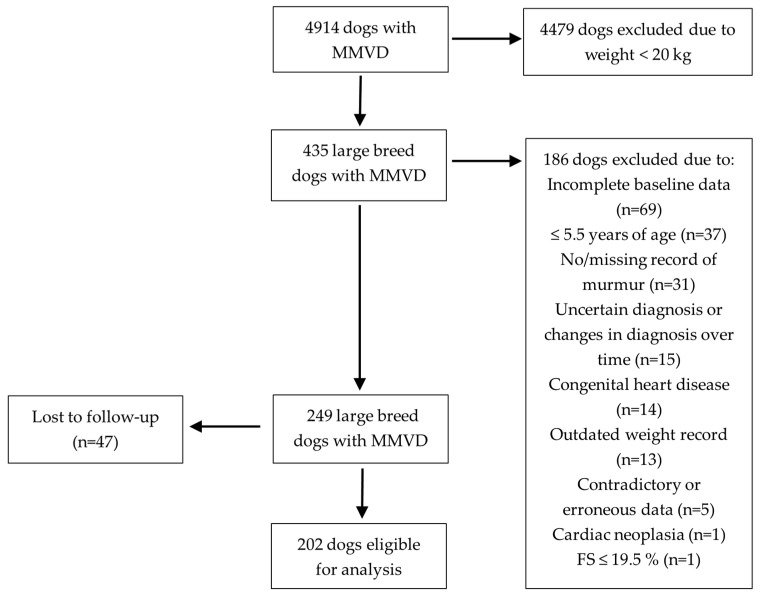
Flowchart showing the inclusion and exclusion process. MMVD, myxomatous mitral valve disease; FS, fractional shortening.

The retrospective review of computerized medical records at AAD and ABR between 2012 and 2021 resulted in 4914 dogs (AAD n = 3249 and ABR n = 1665) diagnosed with MMVD. Four-hundred-and-thirty-five (8.9%) dogs weighed 20 kg or more. Of those, 186 dogs (42.8%) were excluded because of incomplete baseline data, young age, no murmur/missing record of murmur, uncertain diagnosis or changes in diagnosis over time, a diagnosis of congenital heart disease, outdated weight record, contradictory or erroneous data, cardiac neoplasia, and low FS. Forty-seven (18.9%) of the remaining 249 dogs were lost to follow up, leaving 202 dogs eligible for analysis (Figure 1).

### 3.1. Baseline Characteristics

One-hundred-and-thirty-six (67.3%) dogs were male and 66 (32.7%) dogs were female. The most common breeds were mixed breed, Dalmatian, English Springer Spaniel, Labrador Retriever, Rhodesian Ridgeback, and Flat-coated Retriever. The remaining 115 dogs were of 47 different breeds (Table 1).

At diagnosis, the mean weight was 28.94 (SD ± 7.70) kg and the mean age was 10.00 (SD ± 2.04) years.

One-hundred-and-fourteen (56.4%) dogs had a soft murmur, 75 (37.1%) dogs had a moderate/loud murmur, and 10 (5.0%) dogs had a thrilling murmur. Three dogs (1.5%) had a non-graded murmur. One-hundred-and-seventy (84.2%) dogs were classified as ACVIM stage B1, 21 (10.4%) dogs as stage B2, and 11 (5.4%) dogs as stage C. No dog was classified as stage D.

At inclusion, nine dogs were on ongoing cardiac treatment. Five (2.5%) of these dogs were on pimobendan, seven (3.5%) dogs were on furosemide, two (1%) dogs were on spironolactone, and one (0.5%) dog was on an angiotensin-converting enzyme (ACE) inhibitor. Three (33.3%) dogs were staged as B1, four (44.4%) dogs as B2, and two (22.2%) dogs as stage C.

Eight (4.0%) dogs comprising three Golden Retrievers, three mixed breed, one Briard, and one Belgian Shepherd Dog presented with atrial fibrillation, with one (12.5%) dog in stage B1, five (62.5%) dogs in stage B2, and two (25%) dogs in stage C. Seven out of eight (87.5%) dogs with atrial fibrillation were male.

### 3.2. Echocardiographic Measurements at the Time of Inclusion

The overall mean LVIDDN and LVIDSN were 1.84 (SD ± 0.31) and 1.11 (SD ± 0.26), respectively. The overall mean LA/Ao and FS were 1.29 (SD ± 0.35) and 35.3 (SD ± 8.10) %, respectively. MVE was recorded for 88 dogs with a mean of 0.82 (SD ± 0.24) m/s. Echocardiographic measurements at the time of inclusion are summarized in Table 2, grouped according to ACVIM stage.

### 3.3. Disease Progression (from One ACVIM Stage to the Next)

**Figure 2:**
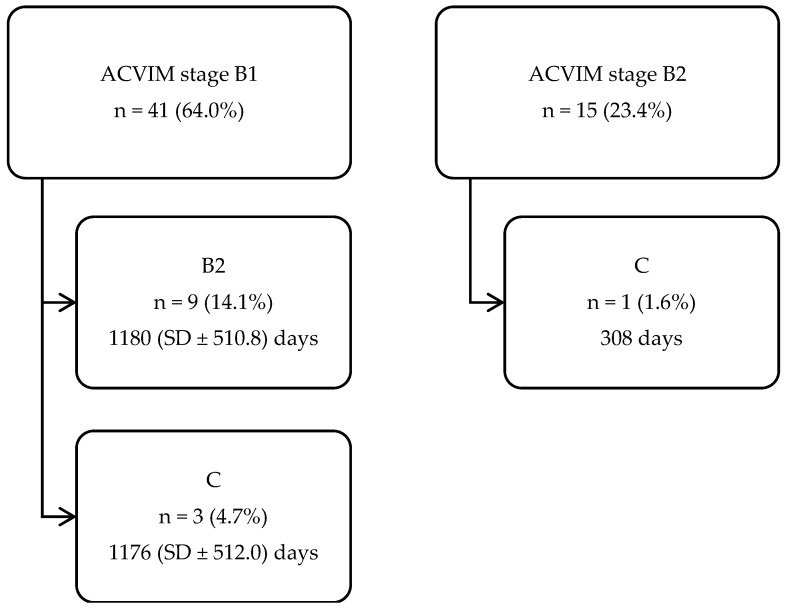
Flowchart showing the number of dogs (n = 64), with a follow-up examination, that progressed from one ACVIM stage to another, including mean follow-up time and SD. ACVIM, American College of Veterinary Internal Medicine; SD, standard deviation.

Sixty-four (31.7%) dogs had a follow-up examination with a mean follow-up time (from diagnosis to last echocardiographic examination) of 710 (SD ± 500.70) days. Fifty-one (79.7%) of these dogs did not progress and 13 (20.3%) dogs progressed from one ACVIM stage to another (Figure 2).

### 3.4. Survival

At the end of the study period, 176 (87.1%) dogs had died, of which 166 (94.3%) were euthanized, and 10 (5.7%) died spontaneously.

In total, 27 (15.3%) deaths were classified as cardiac-related, of which 4 (14.8%) died suddenly. Three out of eight (37.5%) dogs with atrial fibrillation were euthanized due to cardiac reasons and none of these dogs suffered a spontaneous or cardiac-related sudden death.

The median survival time (MST) for the entire population of included dogs, regardless of cause of death, was 716 (CI 560.3–871.7) days. The MST for all-cause mortality was 800 (CI 686.7–913.3) days for stage B1 dogs, 274 (CI 93.1–454.9) days for stage B2 dogs, and 184 (CI 56.7–311.3) days for stage C dogs.

**Figure 3:**
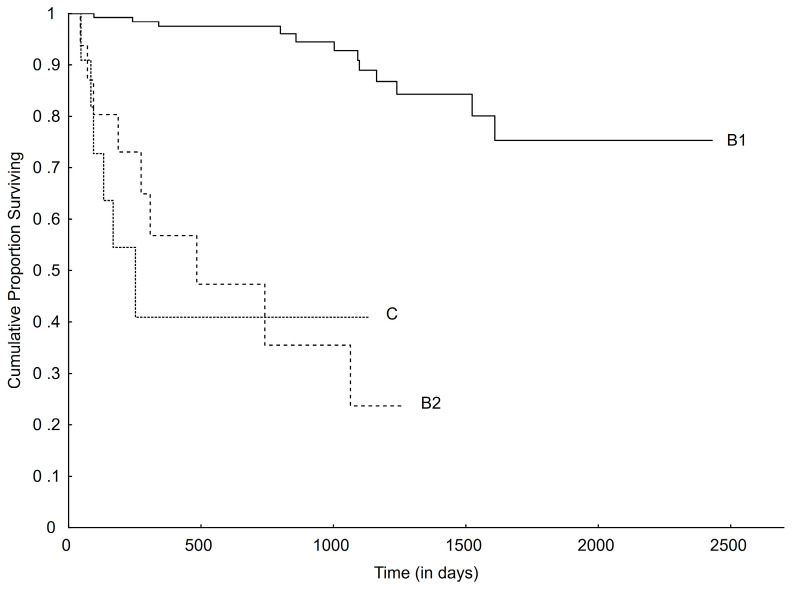
Kaplan–Meier curve plotting survival from initial diagnosis to cardiac-related death for different ACVIM stages (overall comparisons, *p* < 0.001). ACVIM, American College of Veterinary Internal Medicine.

The MST for cardiac-related death, for the entire cohort, could not be calculated (because survival was greater than 50% at the last timepoint). The same was true for stage B1 dogs. For stage B2 and C dogs, the MST for cardiac-related death were 484 (CI 0.0–1050.5) and 252 (CI 83.1–420.9) days, respectively (Figure 3).

**Figure 4:**
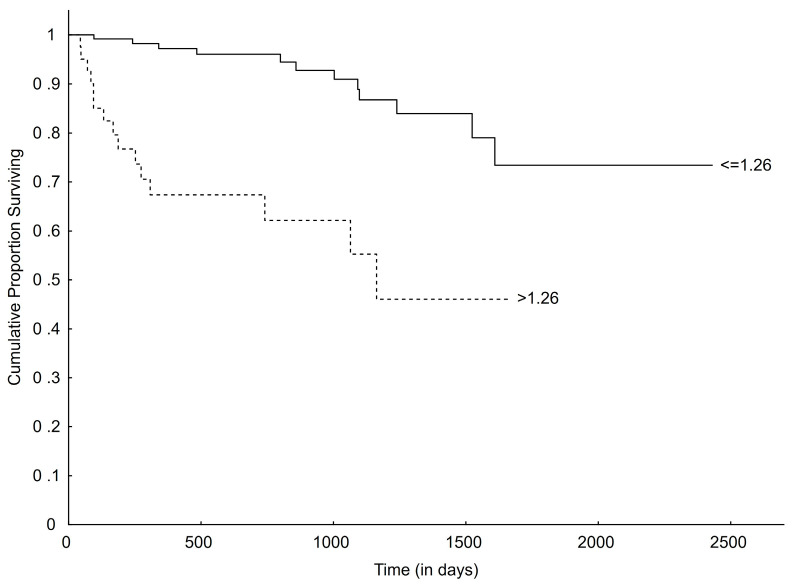
Kaplan–Meier curve plotting survival from time of initial diagnosis to time of cardiac-related death for dogs with normal (≤1.26) and increased (>1.26) LVIDSN (overall comparison, *p* < 0.001). LVIDSN, left ventricular internal diameter in systole normalized.

Considering LVIDSN, dogs were subdivided into two groups according to established upper normal reference, ≤1.26 and >1.26 [26]. Dogs with a normal LVIDSN (≤1.26) had a longer MST (799 days; CI 674.9–923.1; *p* = 0.002) for all-cause mortality than dogs with an increased LVIDSN (>1.26) (444 days; CI 125.2–762.8). The same was true for cardiac-related death, where dogs with a normal LVIDSN (≤1.26) did not reach MST and dogs with an increased LVIDSN (>1.26) had a significantly shorter MST of 1163 days (*p* < 0.001) (Figure 4).

Eight of the dogs (88.9%), with an ongoing cardiac treatment at inclusion, had died at the end of study of which four (50.0%) suffered a cardiac-related death. Median survival time, for both all-cause mortality (*p* < 0.001) and cardiac-related death (*p* < 0.001), was significantly shorter for dogs with pre-existing treatment.

When all IV used in this study were modelled alone, age, LVIDDN, LVIDSN, LA/Ao, being staged as B2 vs. B1, being staged as C vs. B1, and LVIDSN > 1.26 were all significantly associated with all-cause mortality (Table 3).

When the IV were modeled alone, age, having a thrilling vs. a soft murmur, having a thrilling vs. a moderate/loud murmur, LVIDDN, LVIDSN, LA/Ao, MVE, being staged as B2 vs. B1, being staged as C vs. B1, and LVIDSN > 1.26 were all significantly associated with cardiac-related death (Table 4).

When the IV were adjusted for age, sex, and bodyweight, LVIDDN, LVIDSN, LA/Ao, being staged as B2 vs. B1, being staged as C vs. B1, and LVIDSN > 1.26 were all significantly associated with all-cause mortality (Table 3).

When the IV were adjusted for age, sex, and bodyweight, having a thrilling vs. a soft murmur, LVIDDN, LVIDSN, LA/Ao, MVE, being staged as B2 vs. B1, being staged as C vs. B1, and LVIDSN > 1.26 were all significantly associated with cardiac-related death (Table 4).

## 4. Discussion

In previous studies on MMVD in dogs, the presented data have been mostly based on small-and-medium-sized dogs alone or in mixed groups with large breed dogs [7,8,9,14,15,17,18,21,29,30,31,32]. With this study, we provide characteristics, estimated survival times and prognostic variables in a large cohort consisting of only large breed dogs with naturally occurring MMVD.

One of the findings in our study is that preclinical MMVD in large breed dogs, specifically those without left atrial and ventricular enlargement (ACVIM stage B1), progresses slowly and is generally a benign disease where most dogs are expected to succumb due to non-cardiac reasons. Median survival time for cardiac-related death in the aforementioned ACVIM group could not be calculated because survival is greater than 50% at the last timepoint. This is consistent with the results from a study by Borgarelli et al. (2012), where they analyzed a group of dogs with varying sizes, including 24% of dogs weighing more than 20 kg [15]. It is also in line with the results from another report where the MST for stage B1 dogs, weighing up to 20 kg, was found to be 2344 days [21].

Stage B2 dogs, on the other hand, had a relatively short median survival time for both all-cause mortality and cardiac-related death with 274 and 484 days, respectively. This is considerably shorter than the reported 855 and 1341 days for all-cause mortality and cardiac-related death, respectively, in a study consisting of smaller dogs (≤20 kg) [21]. This difference is also evident when comparing our results with the reported MST in the EPIC (≥4.1 and ≤15 kg) and the DELAY (≥2.5 kg and ≤20 kg) studies [8,20]. For stage C dogs, the MST of 252 days for cardiac-related death is very close to the 267 days reported in the QUEST study, where they only included dogs that weighed between 5 and 20 kg [9].

It seems that large breed dogs in stage B1 are unlikely to die in relation to their heart disease. This is in contrast to stage B2 and C dogs, where the adjusted hazard ratios indicate a 12- and 18-fold increase in risk of suffering a cardiac-related death compared to B1 dogs, respectively.

There are several possible explanations for the shown difference in survival times between the large breed dogs in our study and the small-and medium-sized dogs in studies by others. This includes (but is not limited to) randomness, study design/data collection, and/or a true difference in the natural history of MMVD between these groups. Randomness may play a part, as we do not have that many data points in the present study, compared to prospective studies like EPIC and DELAY. If more dogs were included, the results may have been different. The retrospective design of this study means that clinical follow-up and echocardiographic examinations were non-standardized on irregular intervals, which also may have affected outcome. A true difference in survival between large dogs and their small-and medium-sized counterparts can also be the case. Speculatively, the development of systolic dysfunction (based on LVIDSN HR) in large breed dogs may affect the natural history including survival. However, the design of the present study does not allow us to determine if this reflects a true cause and effect relationship.

Given the apparent high risk of cardiac-related death in large breed dogs affected by MMVD with secondary heart changes, careful and frequent monitoring seems prudent (as suggested by some panelists in the ACVIM consensus guidelines) [6].

More than 50 different dog breeds were included in our dataset of large breed dogs with MMVD (Table 1). Breeds like Dalmatian, English Springer Spaniel, Labrador Retriever, Rhodesian Ridgeback, Flat-coated Retriever, and mixed breed dogs were the most common ones. Some of these breeds have been reported to be affected by MMVD in other studies as well, although a thorough comparison is not possible as most studies only report the most common breeds included and not an exhaustive list of breeds [1,5,7,14,15,16]. We cannot conclude from our data whether some large breeds are more commonly affected than others. The presented distribution might just be a result of breed popularity in the represented geographic region.

Our study found that male dogs were overrepresented, with a ratio of 2:1, which is comparable with other reports in both large and small breeds [7,8,15,21]. In addition, sex was not associated with outcome in this study, which also is in line with previously reported data [2,21].

Interestingly, murmur grade has been found to be a prognostic indicator in some but not all studies [7,17]. In our study, having a thrilling vs. a soft murmur turned out to be a negative prognostic indicator of cardiac-related death, but not all-cause mortality, when adjusted for age, sex, and bodyweight. Having a moderate/loud vs. a soft murmur or a thrilling vs. a moderate/loud murmur, on the other hand, were not associated with a difference in cardiac-related outcome.

Increased left atrial and ventricular size have been shown to be prognostic factors related to outcome in several studies [7,8,9,15,16,21,30,32]. This holds true for this study as well, where LVIDDN, LVIDSN and LA/Ao all were significantly associated with both cardiac-related death and all-cause mortality.

It is often stated that MMVD in large breed dogs may include systolic dysfunction more often than in small breed dogs [6,14]. Considering indices of left ventricular systolic function, two variables were analyzed in this study: FS and LVIDSN. LVIDSN was significantly associated with a negative outcome for all-cause mortality and cardiac-related death in both the unadjusted and adjusted analysis. In addition, dogs with a LVIDSN above the reported normal reference range (>1.26), indicating systolic dysfunction, had a significantly shorter median survival time compared to dogs with a normal LVIDSN [26]. These results are in contrast with another study where LVIDSN was not associated with outcome [31].

It has been found that large breed dogs with and without MMVD present with a lower FS than small breed dogs [14,33]. From the data in the present study, one can see that FS in all ACVIM stages have a mean around or below 35%. This result is in line with previously presented data on large breed dogs and lower than reported in groups consisting of only small- and medium-sized dogs [8,14,32,33]. However, FS was neither associated with all-cause mortality nor cardiac-related death. This may be due to the theory that systolic contraction in large breed dogs is more likely to be directed in the long axis, rather than in the short axis like small breed dogs, which in turn reduces FS without necessarily being a result of true systolic dysfunction [33]. Another possibility is that in MMVD, left-ventricular dimensions in diastole and systole increase in parallel, which leaves FS unchanged despite the presence of myocardial failure. Borgarelli et al. (2007) found that both small and large breed dogs affected by MMVD present with increased FS compared to unaffected dogs, which most likely is a result of increased preload, reduced afterload, and thereby, hyperdynamic left ventricular movement [33]. Studies have demonstrated that an increased, rather than a decreased, fractional shortening is associated with a negative outcome as this may indicate a more severe mitral regurgitation [8,16].

MVE turned out to be significantly associated with cardiac-related death in both the unadjusted and the adjusted analysis, which is in line with other studies [7,15,19,30,32].

At presentation, atrial fibrillation was diagnosed in 4% of the dogs included in this study, which is very similar to the 4.1% reported by Borgarelli et al. (2004) for dogs weighing less than 15 kg [14]. In the same study, they found a significantly higher proportion of AF in GSD (31%) [14]. Only four GSD were included in the present study and none of them presented with atrial fibrillation. With such a low number of dogs included from a certain breed, one can neither confirm nor discard previous findings.

Sudden cardiac death (SCD) has been reported to be more prevalent in dogs with atrial fibrillation than in dogs without, with a prevalence of 14.8% and 5.5%, respectively [28]. In the present study, no dog with atrial fibrillation suffered spontaneous or SCD and only 5.7% of all dogs included experienced a spontaneous death, which is more in line with the control group in the aforementioned study. This may be explained by the fact that the study by Borgeat et al. (2021) included dogs with atrial fibrillation, regardless of underlying structural heart disease, including dilated cardiomyopathy, arrhythmogenic cardiomyopathy and congenital heart disease, which are diseases that may be more prone to SCD than MMVD. Mitral valve disease accounted for only 26.7% of the included atrial fibrillation cases, which may explain the difference compared to a group of only MMVD dogs like ours [28].

Interestingly three out of eight (37.5%) dogs with atrial fibrillation were Golden Retrievers, a breed that made up only 2.5% of all dogs included in this study. Atrial fibrillation has previously been reported in Golden Retrievers, both with and without detectable structural heart disease [28,34]. From our data it is impossible to draw any conclusions as to whether Golden Retrievers with MMVD develop atrial fibrillation more often than other breeds, or if they are prone to develop a primary arrhythmia like lone atrial fibrillation and in addition, MMVD.

Seven out of eight (87.5%) dogs with atrial fibrillation were male, which is in line with previous reports where a similar sex predisposition was shown [28,34,35,36].

At initial presentation, only nine dogs received some kind of non-standardized cardiac treatment. When compared to dogs without pre-existing treatment, this smaller group had significantly shorter survival times. However, it is important to note that this group included dogs with more advanced disease stages, and a larger proportion of them experienced cardiac-related deaths. From our data, with such a small number of dogs receiving treatment at inclusion in a non-standardized manner, it is very difficult to draw any conclusions or analyze this further.

### Limitations

Retrospective studies like this one are always associated with inherent limitations and weaknesses. Included cases have been diagnosed and managed by the discretion of the attending veterinarians, albeit experienced, in a non-standardized manner. Treatment regimens may have differed between clinicians and over time as the study period of the present study spanned over 10 years. Some veterinarians may have recommended, and some owners might have chosen, to euthanize their dog instead of starting treatment for congestive heart failure or arrhythmia, which may have influenced the outcome. In addition, 69 dogs were excluded due to incomplete baseline data, which also may have affected the results. The retrospective nature of this study makes it difficult to exclude comorbidities that might have affected outcome and echocardiographic variables.

Dilated cardiomyopathy phenotype (DCM) may be difficult to completely rule out in large breed dogs with MMVD and concurrent evidence of systolic dysfunction as large breed dogs with MMVD can present without valvular changes and thereby resemble DCM [14]. To try to exclude dogs with DCM from our cohort, one exclusion criterion was a FS ≤ 19.5%, as lower values may be more supportive of the diagnosis of DCM [37].

The low number of dogs in this study that progressed into a more advanced disease stage increases the uncertainty and the difficulties of drawing conclusions about disease progression.

In addition, dogs included in this study were diagnosed and managed at two of the largest small-animal hospitals in Sweden, which offer both primary and referral care. Hence, this group of dogs may or may not be representative of the general population of dogs, or comparable with dogs included in other studies. However, this can be an advantage as well, since many studies on MMVD in dogs only include patients from referral clinics, which may comprise patients with more advanced disease that do not respond to conventional management and thereby comprise a selected group of dogs with a different set of characteristics and outcome.

Lastly, this study lacks a matched control group of small-and medium-sized dogs for comparative analysis. This would have given a better opportunity for comparison of survival times and prognostic variables between size-based groups. Prospective studies with matched control groups and well-designed inclusion and exclusion criteria are needed to better characterize MMVD in large breed dogs and to overcome the weaknesses of this study.

## 5. Conclusions

Despite the limitations in our study, we have found that the frequency of cardiac-related death is low in large breed dogs with stage B1 MMVD. We also found that increased left atrial and ventricular size, evidence of systolic dysfunction, a thrilling murmur, and increased early trans-mitral peak velocity were predictors of cardiac-related death. Our data also suggest that the risk of a negative outcome increases profoundly when large breed dogs advance from ACVIM stage B1 into stage B2 or C.

## Figures and Tables

**Table 1:** Breed distribution of included dogs.

Breed	Number of Dogs	Percent (%)
Afghan Hound	2	1.0
Airedale Terrier	7	3.5
American Staffordshire Terrier	2	1.0
Australian Kelpie	5	2.5
Australian Shepherd	5	2.5
Australian Stock Dog	1	0.5
Basset Fauve De Bretagne	1	0.5
Basset Hound	3	1.5
Bavarian Mountain Scent Hound	1	0.5
Beagle	2	1.0
Bearded Collie	1	0.5
Beauce Sheepdog	1	0.5
Border Collie	5	2.5
Boxer	2	1.0
Briard	1	0.5
Bull Terrier	2	1.0
Italian Cane Corso	1	0.5
Dalmatian	9	4.5
Doberman	2	1.0
Bulldog	1	0.5
English Springer Spaniel	9	4.5
Field Spaniel	4	2.0
Finnish Lapponian Dog	1	0.5
Flat-coated Retriever	8	4.0
German Shepherd Dog	4	2.0
Giant Schnauzer	3	1.5
Golden Retriever	5	2.5
Belgian Shepherd Dog	1	0.5
Hamiltonstövare	1	0.5
Hovawart	3	1.5
Irish Red Setter	4	2.0
Irish Soft Coated Wheaten Terrier	1	0.5
Labrador Retriever	9	4.5
Romagna Water Dog	2	1.0
Mixed Breed Dog	43	21.3
Novia Scotia Duck Tolling Retriever	3	1.5
Old Danish Pointing Dog	1	0.5
Petit Basset Griffon Vendéen	1	0.5
Pharaoh Dog	1	0.5
Poodle	7	3.5
Rhodesian Ridgeback	9	4.5
Rottweiler	3	1.5
Samoyed	1	0.5
Schnauzer	3	1.5
Kleiner Münsterländer	2	1.0
Spanish Water Dog	1	0.5
Staffordshire Bull Terrier	2	1.0
Thai Ridgeback	1	0.5
Hungarian Short-haired Pointer (Vizsla)	4	2.0
German Pointing Dog	5	2.5
Weimaraner	2	1.0
Welsh Springer Spaniel	3	1.5
Whippet	1	0.5
Total	202	100

**Table 2:** Echocardiographic baseline characteristics for different ACVIM stage groups.

ACVIM Stage		Age (Years)	Bodyweight (kg)	LVIDDN	LVIDSN	LA/Ao	FS (%)	MVE (m/s)
B1 (n = 170)								n = 79
	Mean	9.91	28.73	1.75	1.05	1.17	35.78	0.77
	SD	2.12	7.83	0.22	0.20	0.16	8.20	0.18
	Min	5.64	20.00	1.08	0.52	0.80	22.20	0.38
	Max	15.13	57.50	2.51	1.79	1.70	64.02	1.59
B2 (n = 21)								n = 6
	Mean	10.67	29.26	2.31	1.47	1.91	32.00	1.20
	SD	1.09	6.28	0.37	0.31	0.35	7.38	0.41
	Min	8.32	21.40	1.90	1.13	1.60	19.65	0.75
	Max	12.25	43.00	3.34	2.44	2.80	46.15	1.72
C (n = 11)								n = 3
	Mean	10.12	31.64	2.28	1.40	2.06	34.34	1.28
	SD	2.00	8.25	0.24	0.23	0.28	6.66	0.10
	Min	7.80	20.50	1.94	1.00	1.80	26.59	1.18
	Max	14.93	45.20	2.70	1.82	2.60	50.00	1.37

ACVIM, American College of Veterinary Internal Medicine; SD, standard deviation; min, minimum; max, maximum; LVIDDN, left ventricular internal diameter in diastole normalized; LVIDSN, left ventricular internal diameter in systole normalized; LA/Ao, left-atrial-to-aortic ratio; FS, fractional shortening; MVE, early trans-mitral peak velocity.

**Table 3:** Unadjusted and adjusted hazard ratios for all-cause mortality.

	Unadjusted		Adjusted ^1^	
Independent Variable	HR [CI]	*p*-Value	HR [CI]	*p*-Value
Age	1.46 [1.34–1.59]	<0.001 *	N/A	N/A
Sex	1.16 [0.84–1.61]	0.372	N/A	N/A
Bodyweight	1.01 [0.98–1.03]	0.651	N/A	N/A
Murmur grade				
Moderate/loud vs. soft	1.37 [1.00–1.86]	0.050	1.23 [0.89–1.68]	0.207
Thrilling vs. soft	1.40 [0.73–2.71]	0.312	1.29 [0.66–2.52]	0.449
Thrilling vs. moderate/loud	1.03 [0.53–2.01]	0.935	1.06 [0.54–2.08]	0.877
LVIDDN (HR for 0.1-unit increment)	1.13 [1.07–1.20]	<0.001 *	1.11 [1.05–1.17]	<0.001 *
LVIDSN (HR for 0.1-unit increment)	1.12 [1.05–1.20]	0.001 *	1.15 [1.08–1.23]	<0.001 *
LA/Ao (HR for 0.1-unit increment)	1.13 [1.08–1.18]	<0.001 *	1.10 [1.05–1.15]	<0.001 *
FS (HR for 10% increment)	1.03 [0.85–1.24]	0.778	0.84 [0.70–1.02]	0.085
MVE (HR for 0.1-unit increment)	1.04 [0.95–1.14]	0.405	0.97 [0.88–1.08]	0.572
ACVIM stage				
Stage B2 vs. B1	2.63 [1.61–4.29]	<0.001 *	1.73 [1.06–2.83]	0.029 *
Stage C vs. B1	3.34 [1.78–6.25]	<0.001 *	2.19 [1.15–4.19]	0.017 *
Stage C vs. B2	1.27 [0.60–2.68]	0.527	1.27 [0.59–2.71]	0.540
LVIDSN > 1.26	1.73 [1.21–2.47]	0.003 *	1.93 [1.34–2.77]	<0.001 *

*, significant; HR, hazard ratio; aHR, adjusted hazard ratio; CI, 95% confidence interval; N/A, not applicable; ACVIM, American College of Veterinary Internal Medicine; LVIDDN, left ventricular internal diameter in diastole normalized; LVIDSN, left ventricular internal diameter in systole normalized; LA/Ao, left-atrial-to-aortic ratio; FS, fractional shortening; MVE, early trans-mitral peak velocity. ^1^ Adjusted for age, sex and bodyweight.

**Table 4:** Unadjusted and adjusted hazard ratios for cardiac-related death.

	Unadjusted		Adjusted ^1^	
Independent Variable	HR [CI]	*p*-Value	HR [CI]	*p*-Value
Age	1.36 [1.10–1.67]	0.004 *	N/A	N/A
Sex	2.63 [0.91–7.62]	0.075	N/A	N/A
Bodyweight	0.97 [0.91–1.02]	0.241	N/A	N/A
Murmur grade				
Moderate/loud vs. soft	2.02 [0.86–4.73]	0.105	1.92 [0.82–4.51]	0.136
Thrilling vs. soft	5.93 [1.98–17.71]	0.001 *	4.81 [1.55–14.93]	0.007 *
Thrilling vs. moderate/loud	2.93 [1.04–8.24]	0.041 *	2.51 [0.86–7.33]	0.092
LVIDDN (HR for 0.1-unit increment)	1.37 [1.24–1.51]	<0.001 *	1.32 [1.20–1.46]	<0.001 *
LVIDSN (HR for 0.1-unit increment)	1.40 [1.25–1.56]	<0.001 *	1.35 [1.21–1.51]	<0.001 *
LA/Ao (HR for 0.1-unit increment)	1.35 [1.25–1.47]	<0.001 *	1.31 [1.21–1.43]	<0.001 *
FS (HR for 10% increment)	0.77 [0.45–1.29]	0.317	0.64 [0.38–1.10]	0.107
MVE (HR for 0.1-unit increment)	1.32 [1.11–1.57]	0.002 *	1.29 [1.08–1.54]	0.005 *
ACVIM stage				
Stage B2 vs. B1	14.01 [5.60–35.04]	<0.001 *	12.20 [4.64–32.08]	<0.001 *
Stage C vs. B1	17.95 [6.26–51.47]	<0.001 *	18.25 [5.45–61.10]	<0.001 *
Stage C vs. B2	1.28 [0.45–3.64]	0.641	1.50 [0.50–4.45]	0.469
LVIDSN >1.26	5.50 [2.56–11.79]	<0.001 *	5.91 [2.71–12.86]	<0.001 *

*, significant; HR, hazard ratio; aHR, adjusted hazard ratio; CI, 95% confidence interval; N/A, not applicable; ACVIM, American College of Veterinary Internal Medicine; LVIDDN, left ventricular internal diameter in diastole normalized; LVIDSN, left ventricular internal diameter in systole normalized; LA/Ao, left-atrial-to-aortic ratio; FS, fractional shortening; MVE, early trans-mitral peak velocity. ^1^ Adjusted for age, sex and bodyweight.

## Data Availability

The data used to generate the results in this manuscript can be made available if requested from the corresponding author. The data are not publicly available due to professional secrecy.
